# Genome-wide identification, evolutionary and expression analyses of LEA gene family in peanut (*Arachis hypogaea* L.)

**DOI:** 10.1186/s12870-022-03462-7

**Published:** 2022-03-30

**Authors:** RuoLan Huang, Dong Xiao, Xin Wang, Jie Zhan, AiQing Wang, LongFei He

**Affiliations:** 1 National Demonstration Center for Experimental Plant Science Education, College of Agriculture, Guangxi University, Nanning, 530004 China; 2 Guangxi Key Laboratory for Agro-Environment and Agro-Product Safety, Nanning, 530004 China; 3 Key Laboratory of Crop Cultivation and Tillage, Guangxi Colleges and Universities, Nanning, 530004 China

**Keywords:** *Arachis hypogaea* L., Late embryogenesis abundant, Expression profiles, Abiotic stress

## Abstract

**Background:**

Late embryogenesis abundant (LEA) proteins are a group of highly hydrophilic glycine-rich proteins, which accumulate in the late stage of seed maturation and are associated with many abiotic stresses. However, few peanut *LEA* genes had been reported, and the research on the number, location, structure, molecular phylogeny and expression of *AhLEA*s was very limited.

**Results:**

In this study, 126 *LEA* genes were identified in the peanut genome through genome-wide analysis and were further divided into eight groups. Sequence analysis showed that most of the *AhLEA*s (85.7%) had no or only one intron. *LEA* genes were randomly distributed on 20 chromosomes. Compared with tandem duplication, segmental duplication played a more critical role in *AhLEA*s amplication, and 93 segmental duplication *AhLEA*s and 5 pairs of tandem duplication genes were identified. Synteny analysis showed that some *AhLEA*s genes come from a common ancestor, and genome rearrangement and translocation occurred among these genomes. Almost all promoters of *LEA*s contain ABRE, MYB recognition sites, MYC recognition sites, and ERE cis-acting elements, suggesting that the *LEA* genes were involved in stress response. Gene transcription analyses revealed that most of the *LEA*s were expressed in the late stages of peanut embryonic development. *LEA3* (AH16G06810.1, AH06G03960.1), and *Dehydrin* (AH07G18700.1, AH17G19710.1) were highly expressed in roots, stems, leaves and flowers. Moreover, 100 *AhLEA*s were involved in response to drought, low-temperature, or Al stresses. Some *LEA*s that were regulated by different abiotic stresses were also regulated by hormones including ABA, brassinolide, ethylene and salicylic acid. Interestingly, *AhLEA*s that were up-regulated by ethylene and salicylic acid showed obvious subfamily preferences. Furthermore, three *AhLEA* genes, *AhLEA1*, *AhLEA3-1*, and *AhLEA3-3*, which were up-regulated by drought, low-temperature, or Al stresses was proved to enhance cold and Al tolerance in yeast, and *AhLEA3-1* enhanced the drought tolerance in yeast.

**Conclusions:**

*AhLEA*s are involved in abiotic stress response, and segmental duplication plays an important role in the evolution and amplification of *AhLEA*s. The genome-wide identification, classification, evolutionary and transcription analyses of the *AhLEA* gene family provide a foundation for further exploring the *LEA* genes’ function in response to abiotic stress in peanuts.

**Supplementary Information:**

The online version contains supplementary material available at 10.1186/s12870-022-03462-7.

## Background

Plant in nature often encounters various abiotic stresses including drought, cold, high temperature, and salinity, which affect growth and development, reduce its yield and survival rate. Plants have evolved many mechanisms to cope with various environmental stresses. It is known that the late embryogenesis abundant (LEA) proteins play important roles in protecting cells under abiotic stresses, and many *LEA*s are induced by cold, drought, salinity, abscisic acid (ABA), and ethylene [1–3]. Moreover, it has been confirmed that *AdDHN1*, a member of the Dehydrin family, can improve the drought resistance of transgenic Arabidopsis, but it is more sensitive to nematodes (Mota et al., 2018), which indicated that some of the *LEA*s may respond to abiotic stress as well as biotic stress.

LEA proteins are highly hydrophilic glycine-rich proteins, which accumulate largely in the later stage of seed maturation and fade away following germination [4, 5]. As water-binding molecules, the role of LEA proteins is enhancing the stability of protein and membrane. Subcellular localization analysis has indicated that LEA proteins are mainly located in nuclear regions and the cytoplasm [6]. LEA proteins have been observed in the roots, leaves, buds, and seedlings, although they mainly appear in seeds of plants [7].

LEA protein families were identified in many plant species by genome-wide identification and analysis, such as *Arabidopsis thaliana* [8]*, Populus trichocarpa* [5]*, Camellia sinensis* [9]*, Brassica napus* [10]*,* and *Triticum aestivum* [11]. During the growth and development of plant, LEA proteins are considered to play important roles. It was reported that *Medicago falcate LEA3* conferred multiple abiotic stress tolerance by involving the protection of catalase activity [12]. A heterologous expression of a barley LEA3 protein gene, *HVA1*, improved tolerance to water stress in rice and wheat [13, 14]. *AtLEA5* protects yeast cells against oxidative stress [15]. *Escherichia coli* can grow in high salt and extreme temperature conditions due to the over-expression of soybean PM2 protein (LEA3) [16, 17]. ABA can regulate the expression of many LEA proteins, and it was proved that the expression of *LEA4* subfamily members was upregulated by exogenous ABA [18].

Peanut is one of the main oils and cash crops all over the world. Peanut is a rainfed crop, but it is sensitive to water deficit stress in the flowering and pegging stages, which would impact the yield of peanuts [19]. Also, Al stress inhibition of growth reduces peanut yield in acid soil [20]. To date, the function of the *LEA* gene family in peanuts has little been reported. In this study, we identified the *LEA*s in peanut and analyzed the structure, evolution, and chromosome location of peanut *LEA*s. Our findings provide a foundation for the evolutionary and functional characterization of *LEA* gene families in peanut and other plant species.

## Results

### Identification and characteristics of *AhLEA* gene in peanut

**Fig. 1:**
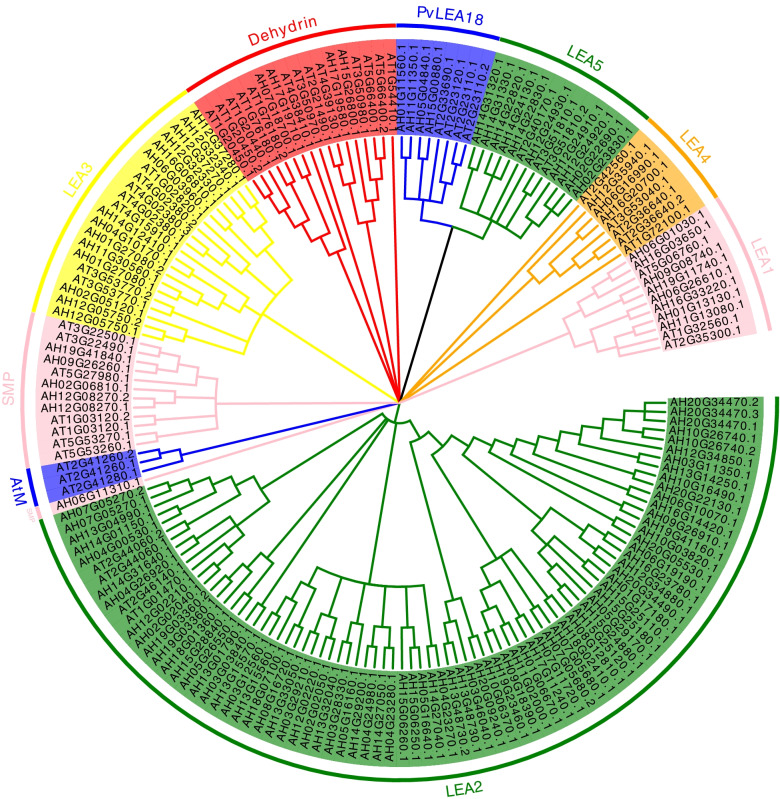
Phylogenetic relationships of the AhLEAs and AtLEAs. The Maximum Likelihood (ML) tree was generated using MEGA7 with 1000 bootstrap replicates. *LEA* gene families are distinguished by different colors

By using the publicly available peanut genome sequence data, the genome-wide identification of *LEA*s in peanuts based on sequence homology with 51 Arabidopsis *LEA*s was performed [21] (Table 1 and Additional file 1: Table S1). Proteins that contain a conserved LEA domain were screened by the NCBI-BLAST online tool. Eventually, 126 *AhLEA*s were identified. All of these genes were grouped with 51 *AtLEA*s by phylogenetic analyses. The *AhLEA*s were classified into eight subfamilies including *LEA1*, *LEA2*, *LEA3*, *LEA4*, *LEA5*, *PvLEA18*, *SMP*, and *Dehydrin* (Fig. 1). The *LEA2* family was the largest, with 78 members. The *LEA3*s and *LEA5*s had 14 and 10 members, respectively. The *LEA1*s had 8 members, *SMP* had 6 members and *PvLEA18* had 4 members. The *LEA4* and *Dehydrin* families had 3 members. The species-specific group (*AtM*) of Arabidopsis was absent in the peanut.

**Table 1:** The classification of LEA proteins in *Arachis hypogaea* is based on Arabidopsis

In this study	IPR ID	Pfam ID	Hundertmark et al. (2008)	Arabidopsis	A. hypogaea
*LEA1*	IPR005513	PF03760	*LEA 1*	7	8
*LEA2*	IPR004864/IPR013990	PF03168	*LEA 2*	3	78
*LEA3*	IPR004926	PF03242	*LEA 3*	4	14
*LEA4*	IPR004238	PF02987	*LEA 4*	18	3
*LEA5*	IPR000389	PF00477	*LEA 5*	2	10
*PvLEA18*	IPR018930	PF10714	*PvLEA18*	3	4
*SMP*	IPR007011	PF04927	*SMP*	6	6
*Dehydrin*	IPR000167	PF00257	*Dehydrin* *AtM*	10 2	3 0

### Chromosomal locations, gene duplication and synteny analysis of the *AhLEA*s

**Fig. 2:**
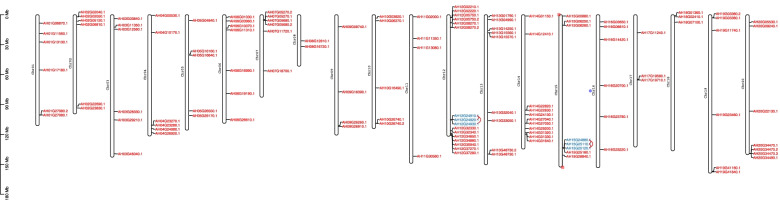
Chromosome distributions of the *AhLEA*s and gene duplication events. Distribution of 126 genes on chromosomes of peanut, the blue words represent pairs of tandem duplication genes

The identified 126 *AhLEA*s were further located on the 20 chromosomes (Fig. 2). The largest number of *AhLEA*s was found on chromosome 12, fourteen genes, followed by chromosome 14 (eleven genes). The lowest loci density was observed on chromosome 8, with only two genes. Eight genes were found located on chromosomes 13 and 15, seven genes on chromosome 6. Five chromosomes (chr1, chr2, chr3, chr4, and chr16) carried six *AhLEA*s and four chromosomes (chr5, chr7, chr19, and chr20) carried five *AhLEA*s. Chromosomes 9, 10, and 11 contained four *AhLEA*s, and chromosomes 17, 18 contained three *AhLEA*s. The *AhLEA*s were distributed unevenly among the 20 chromosomes in peanut. All chromosomes contained the *LEA2*s, and all of the *LEA*s on chromosomes 3, 8, 10, 13, 18, and 20 belonged to the *LEA2*s. Chromosomal location analysis of *AhLEA*s indicated that eight subfamilies were distributed unevenly in the genome (Fig. 2).

**Fig. 3:**
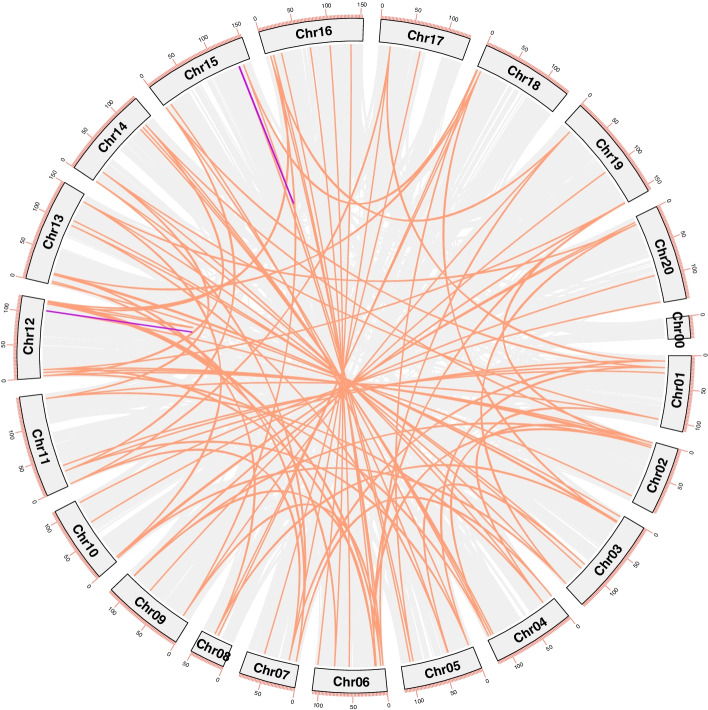
Duplication analysis of 126 *AhLEA*s. The rectangle on the outer ring represents peanut chromosome 00–20. The purple line on chromosomes 12, 15 represents tandem duplication gene pairs, and light orange lines on chromosomes represent segmental duplication gene pairs

**Fig. 4:**
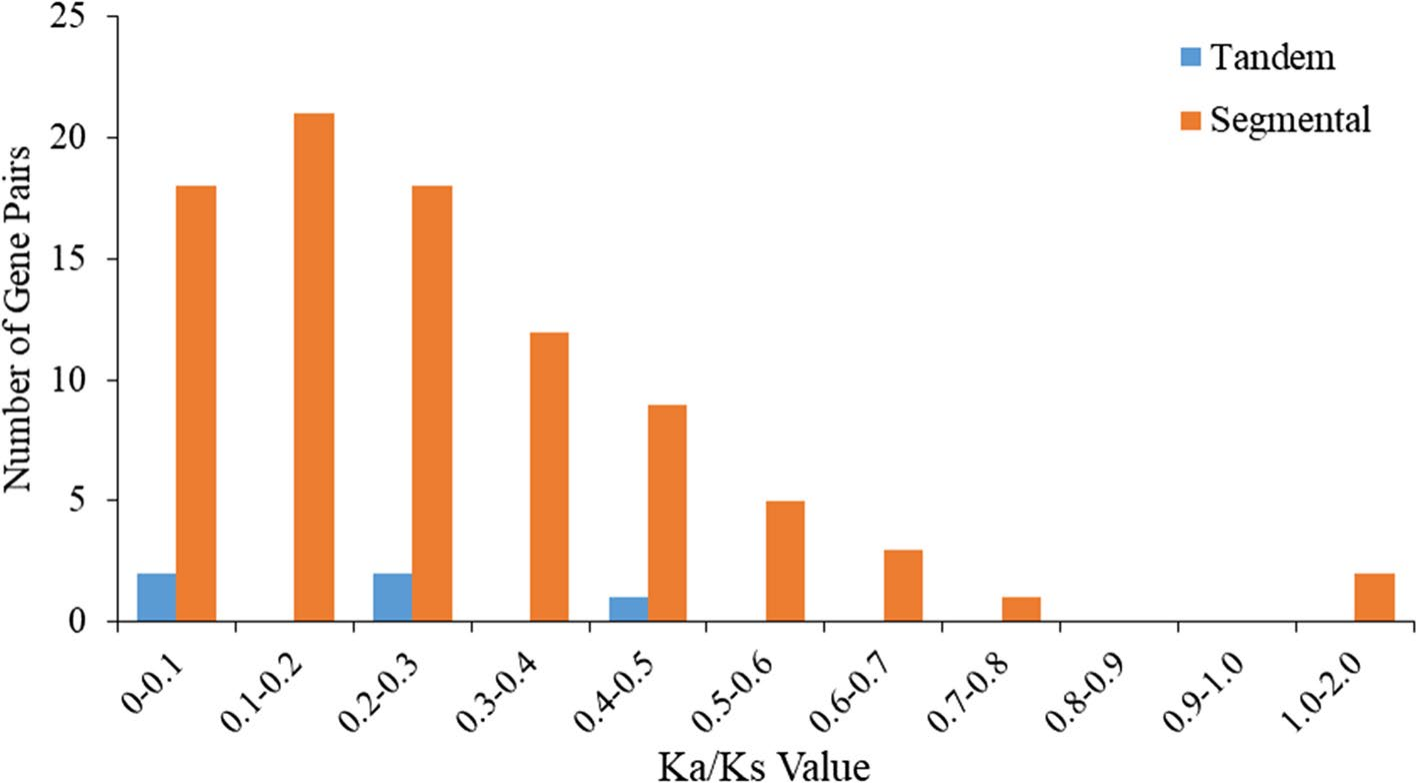
The distribution of Ka/Ks values in all tandem and segmental duplicated *AhLEA*s

**Fig. 5:**
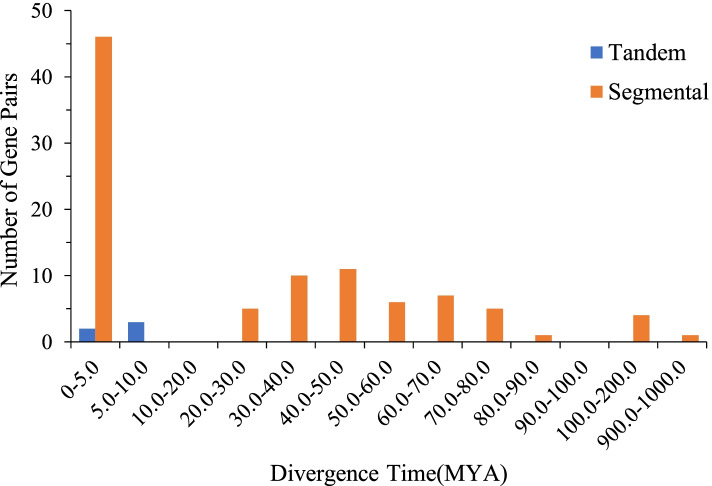
The distribution of divergence time (MYA) in all tandem and segmental duplicated *AhLEA*s

The generation and evolution of gene families may be caused by tandem duplication and segmental duplication [22, 23]. To investigate the evolutionary relationships of the *AhLEA* gene family, we analyzed the duplication events of *AhLEA*s (Fig. 3). In this study, five pairs of tandem duplication and 93 pairs of segmental duplication were identified (Fig. 3, Additional file 1: Table S2). Five tandem duplication pairs belong to the *LEA5*s and *LEA2*s, and were located on chromosomes 12 and 15. The segmental duplication genes were mainly distributed on chromosome 12. All members of the *LEA1*s, *SMP*s, and *PvLEA18*s were segmental duplication genes, followed by *LEA2*s (79.5%) and *LEA3*s (71.4%). The Ka/Ks values of all the tandem duplication gene pairs were less than 1. Except for four segmental duplication gene pairs whose Ka/Ks values could not be calculated, the Ka/Ks values of the most segmental duplication gene pairs were less than 1, and only two pairs (2.2%) were more than 1 (Fig. 4, Additional file 1: Table S2). The divergence time of tandem duplication events was mainly 0–10 million years ago (MYA), and 49.5% (46/93) of segmental duplication events occurred between 0–5 MYA (Fig. 5, Additional file 1: Table S2).

**Fig. 6:**
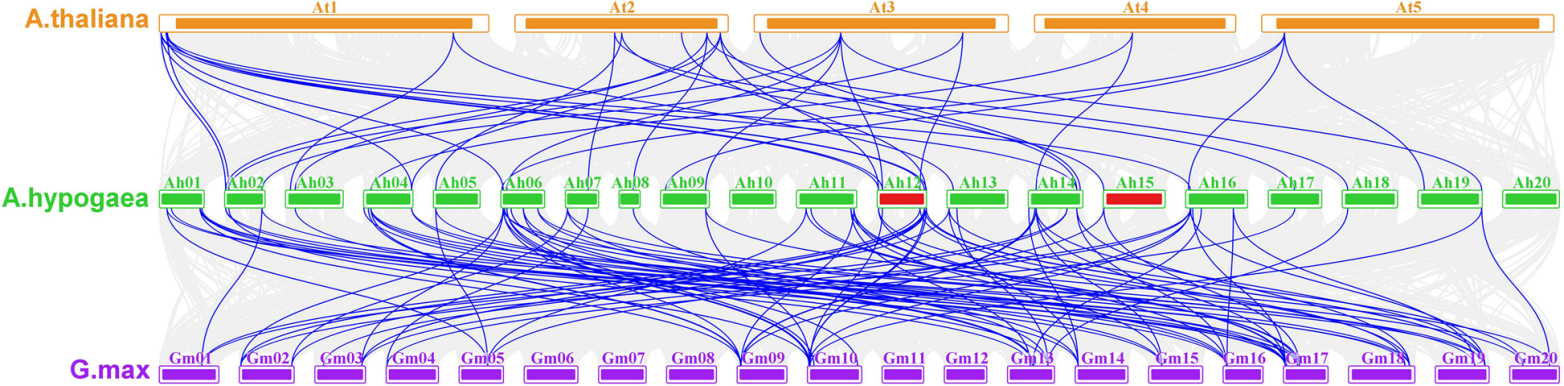
Synteny analyses of *AhLEA*s to Arabidopsis and G. max. Gray lines in the background indicate collinear blocks within peanut and Arabidopsis, soybean genomes, while blue lines highlight syntenic *LEA* gene pairs, Red chromosome blocks represent tandem duplicated genes

To explore the evolutionary process of the peanut *LEA* genes, we performed synteny analysis among peanut, Arabidopsis, and soybean. *AhLEA*s showed a more syntenic to soybean than Arabidopsis (Fig. 6, Additional file 1: Table S3). Thirteen orthologous pairs exhibited single gene correspondences between peanut and Arabidopsis, and five orthologous pairs exhibited single gene correspondences peanut and soybean. Five *AhLEA*s were associated with multiple *AtLEA*s, and fourteen *AhLEA*s were associated with *GmLEA*s. Additionally, there were nine cases that peanut segmental duplications that corresponded to a single Arabidopsis gene, and eleven cases that *AhLEA*s corresponded to a single soybean gene. Finally, some genes showed more-to-more correspondence.

### Analysis of gene structure and protein motifs of *LEA*s in peanut

**Fig. 7:**
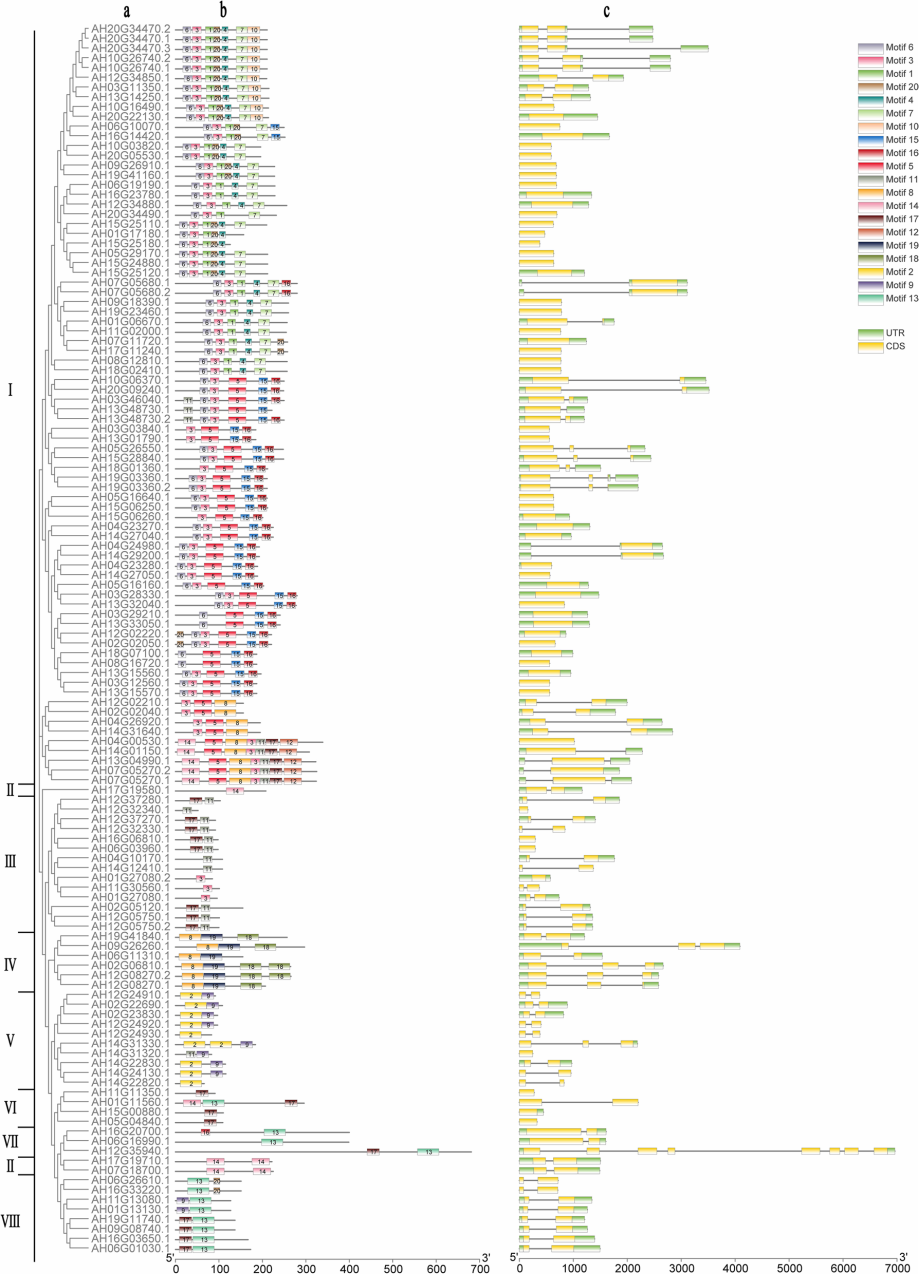
Phylogenetic relationships, gene structures, and compositions of the conserved protein motifs of the *AhLEA*s.I: *LEA2*; II: *Dehydrin*; III: *LEA3*; IV: *SMP*; V: *LEA5*; VI: *PvLEA18*; VII: *LEA4*; VIII: *LEA1*; a: Phylogenetic relationships, b: conversed motif, c: UTR–CDS organization, black lines represent intron

To examine the structural characteristics of *AhLEA*s, an unrooted phylogenetic tree that combines the UTR-CDS structures and motifs were constructed based on the full lengths of the 126 peanut *LEA* genes sequence by using the Maximum-Likelihood method (Fig. 7). The majority of the *AhLEA*s contained zero or one intron, with 55 and 53, respectively. Sixteen genes had two introns. One gene, AH19G03360.1, contained three introns, and one gene, AH12G35940.1, contained seven introns. All the *LEA1*s and *Dehydrin*s contained only one intron, and the main members of the *LEA3* and *LEA5* subfamilies had one intron. The majority of the *LEA2*s had no intron. To identify the conserved protein motifs, the MEME (http://meme-suite.org/tools/meme) online software was used to predict putative motifs of these proteins, with a maximum number of the different motifs at 20. Motif analysis indicated that members of each subfamily had the group-specific conserved domain, and *AhLEA*s with closer evolutionary relationships had more similar motif numbers. MEME analysis revealed that most *AhLEA*s contained motif 3 and all the *LEA4*s and *LEA1*s had motif 13. The *LEA2*s had the greatest number of motifs, which were 7 motifs, while other subfamily members had 1 to 4 motifs.

### Analysis of cis-acting elements in promoters of *AhLEA*s

**Fig. 8:**
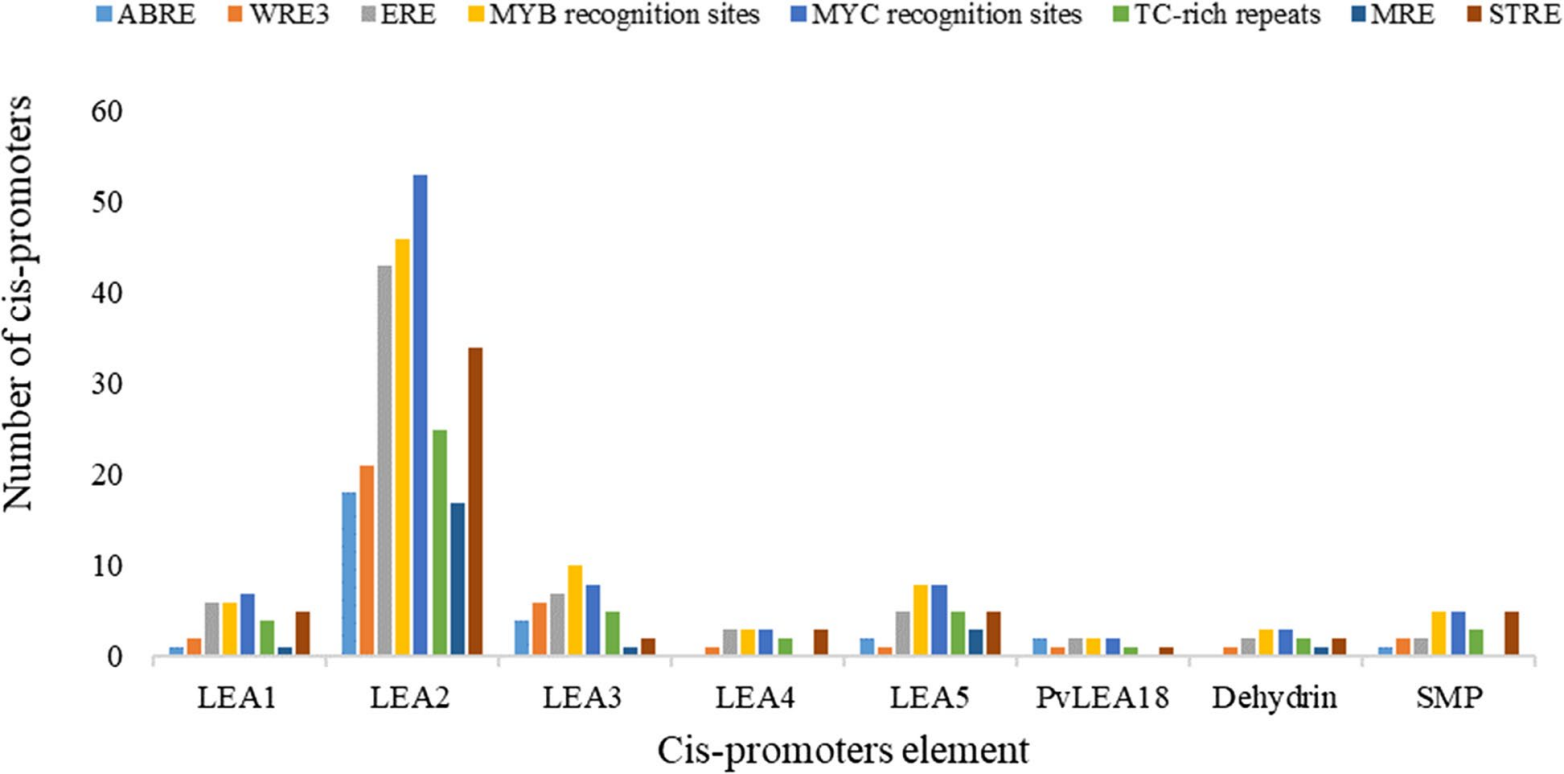
Distribution of major abiotic stress-responsive cis-elements in the promoter sequences of the 126 *AhLEA*s

To investigate the cis-acting elements of *AhLEA*s, 2 kb region upstream of the translation initiation sites of all the *LEA* genes were obtained from the peanut genome database. Many cis-acting regulatory elements that may be involved in the response to environmental stresses in plant, including ABRE, WRE3, ERE, MYB recognition sites, MYC recognition sites, TC-rich repeats, STRE, and MRE, were detected (Fig. 8). The promoter of subfamily *LEA2* contained the most cis-acting elements, followed by subfamily *LEA3*, *LEA5*, and *LEA1*. The promoter of subfamily *LEA4*, *SMP*, *PvLEA18*, and *Dehydrin* contained the least elements. Among the identified cis-acting elements, ABRE (22.2%), ERE (55.6%), MYB recognition sites (65.9%), and MYC recognition sites (70.6%) cis-acting elements were over-represented.

### Expression profiles of *AhLEA*s in different tissues and at different stages of embryo development

**Fig. 9:**
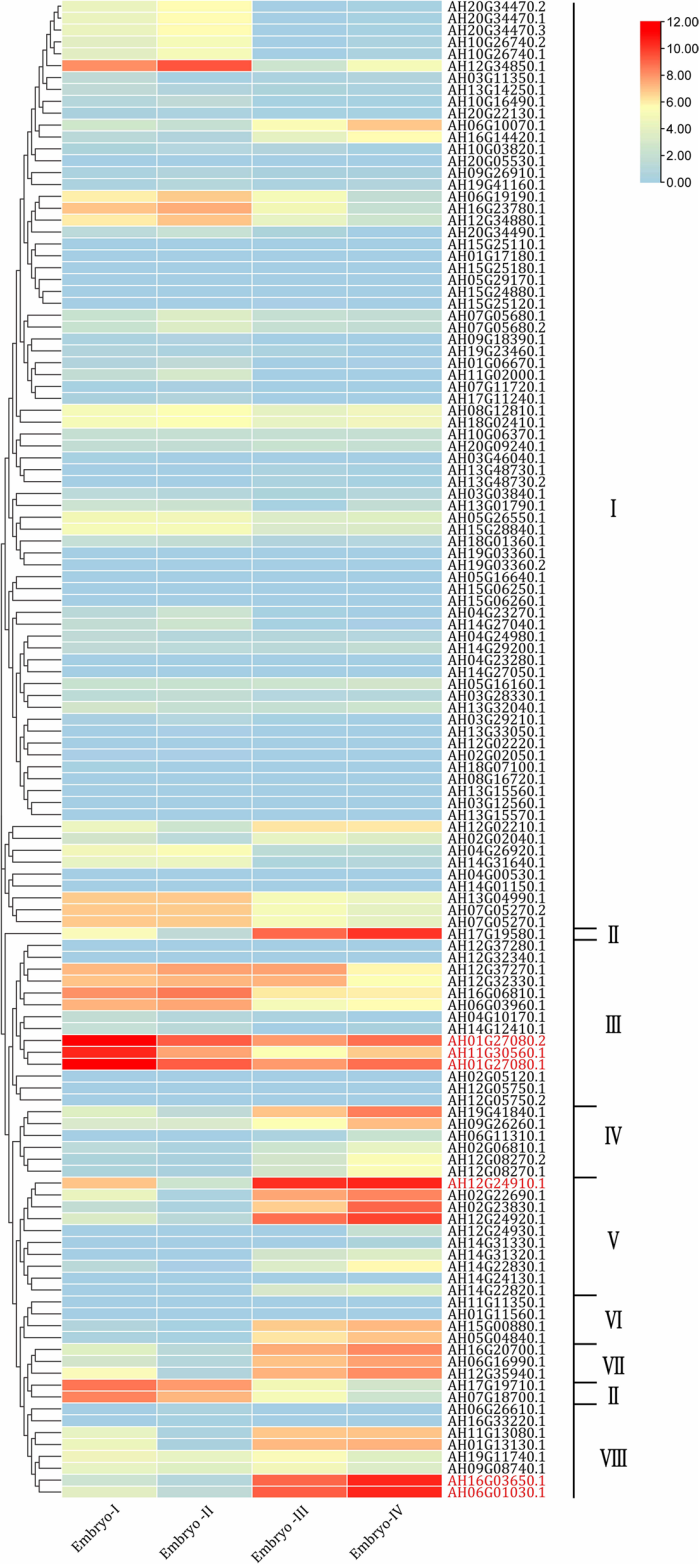
A heatmap showing the hierarchical clustering of the expression levels of the 126 *AhLEA*s in the four embryo periods in peanut. I: *LEA2*; II: *Dehydrin*; III: *LEA3*; IV: *SMP*; V: *LEA5*; VI: *PvLEA18*; VII: *LEA4*; VIII: *LEA1*

To investigate the expression profiles of *AhLEA*s across different stages of embryo development and different tissues, the transcriptomic data of a cultivated variety (*A. hypogaea* L.) in gene bank were further scrutinized (http://peanutgr.fafu.edu.cn/Transcriptome.php) (Fig. 9, Additional file 1: Table S4). Not all *AhLEA*s were expressed at the four embryo development stages. Meanwhile, twenty-seven genes were not detected at any tested stages. Sixty-eight *LEA*s had different transcription levels among the four stages. In the early embryo development stages, most *LEA3*s were up-regulated. Among them, three *LEA3*s (AH01G27080.1, AH01G27080.2, and AH11G30560.1) exhibited very high transcription levels in the early stages, which showed up to tenfold higher than those in the late stages. Nevertheless, *AhLEA1*s, *AhLEA4*s, and *AhLEA5*s were up-regulated mainly in the late stages. Four genes including two *LEA5*s (AH12G24910.1 and AH12G24920.1) and two *LEA1*s (AH06G01030.1 and AH16G03650.1) exhibited very high transcription levels in the late stages. Two genes of the *Dehydrin*s expressed at a high level in stages I, and II, while another *Dehydrin* (AH17G19580.1) expressed at a high level in stages III and IV. The transcription of most *AhLEA2*s was not changed among the four tested stages, while the transcription level of one *LEA2* (AH12G34850.1) in the early stages showed up to 26-fold higher than those in the later stages.

**Fig. 10:**
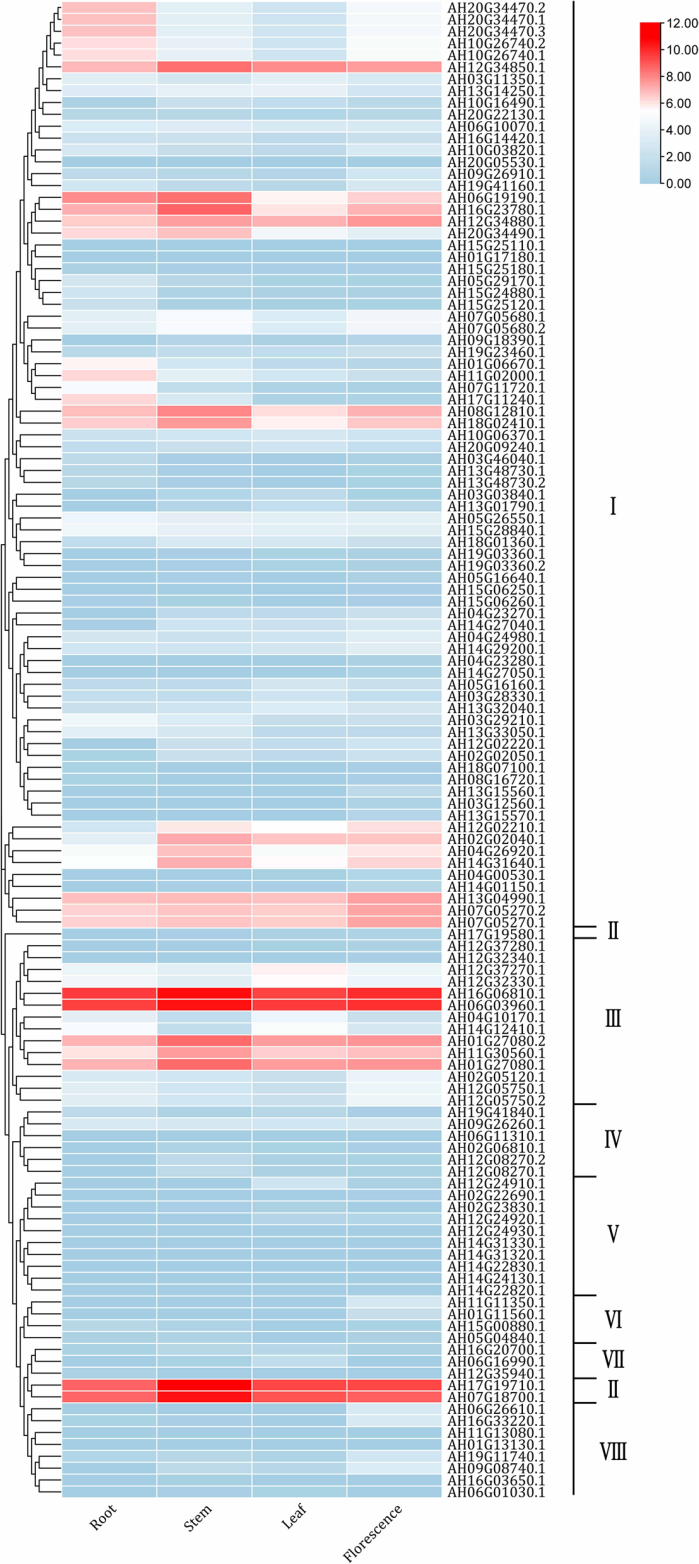
A heatmap showing the hierarchical clustering of the expression levels of the 126 *AhLEA*s in the roots, stems, leaves, and flowers of peanut. I: *LEA2*; II: *Dehydrin*; III: *LEA3*; IV: *SMP*; V: *LEA5*; VI: *PvLEA18*; VII: *LEA4*; VIII: *LEA1*

As shown in Fig. 10, the expression profiles of eight subfamilies, including *LEA1*s, *LEA2*s*, LEA3*s, *LEA4*s *LEA5*s, *SMP*s, *PvLEA18*s, and *Dehydrin*s, were similar in roots, stems, leaves, and flowers. Among them, the members of *LEA2*s*, LEA3*s, and *Dehydrin*s were expressed at a high level in all four tissues. Twenty-four *LEA*s were highly expressed in roots, 21 in stems, 15 in leaves, and 20 in flowers. Two *Dehydrin*s (AH07G18700.1 and AH17G19710.1) and two *LEA3*s (AH16G06810.1 and AH06G03960.1) had the highest transcription levels in the stem (Additional file 1: Table S5).

### Expression profiles of *AhLEA*s in response to drought and low-temperature stresses.

**Fig. 11:**
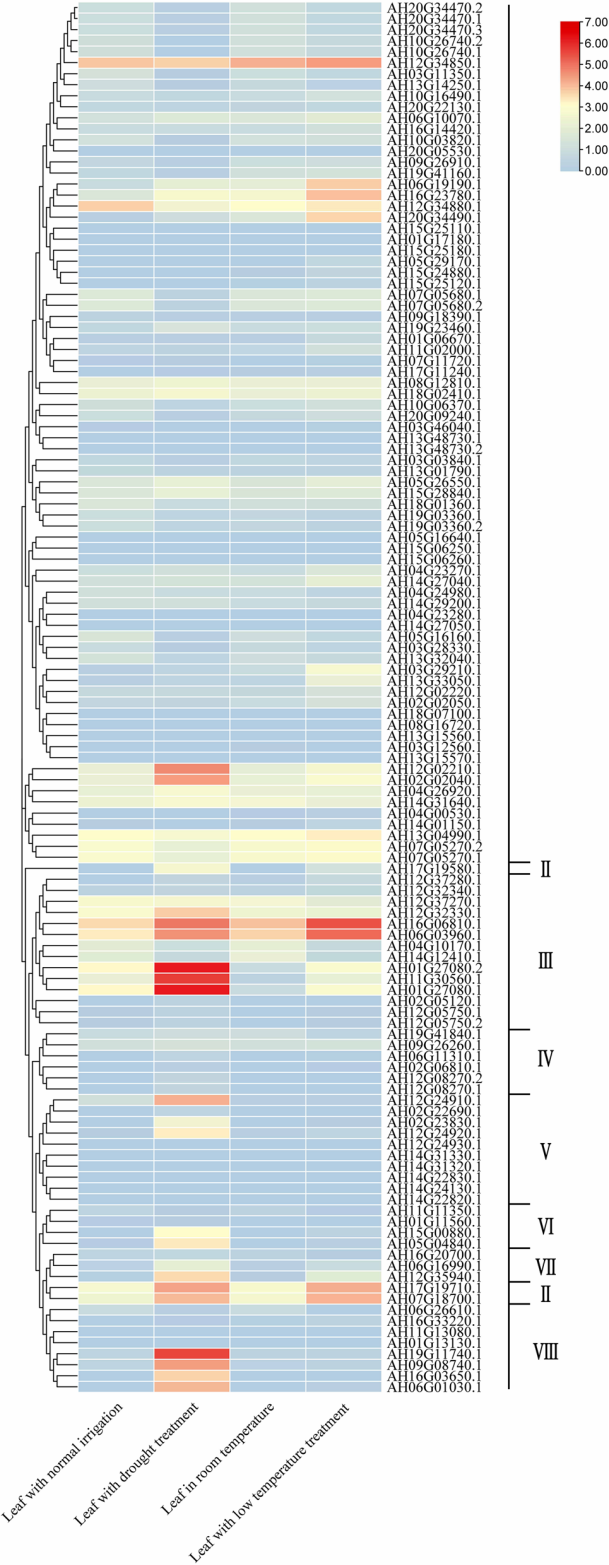
Expression profiles of the AhLEAs in peanut. Dynamic expression profiles of *AhLEA*s drought and low-temperature treatments using heatmap of hierarchical clustering. I: *LEA2*; II: *Dehydrin*; III: *LEA3*; IV: *SMP*; V: *LEA5*; VI: *PvLEA18*; VII: *LEA4*; VIII: *LEA1*

To investigate the transcriptional changes of the *AhLEA*s under cold and drought stresses, the expression profiles of these genes were examined by using transcriptomic data (Fig. 11). Under drought treatment, 28.6% (36 out of 126) of the *AhLEA*s were up-regulated more than twofold compared with the control, while the transcription levels of 21.4% (27 out of 126) genes were down-regulated more than twofold. Among the 27 genes that down-regulated more than twofold, 24 genes belonged to the *LEA2* subfamily. Two *LEA3*s (AH01G27080.1, and AH01G27080.2) showed the highest transcription levels under drought stress (Additional file 1: Table S6).

Under low-temperature treatment, 28.6% (36 out of 126) of the *AhLEA*s were up-regulated more than twofold compared with the control, while the transcription levels of 14.3% (18 out of 126) genes were down-regulated more than twofold. It was found that 21 genes of *LEA2*s were up-regulated and 11 genes were down-regulated. It is noteworthy that all *Dehydrin*s were up-regulated under drought and low-temperature stresses. Interestingly, the genes expressed the highest under low-temperature stress were also two *LEA3* subfamily genes (AH16G06810.1, AH06G03960.1) (Fig. 11: Additional file 1: Table S6).

### Expression profiles of *AhLEA* genes in response to hormone

**Fig. 12:**
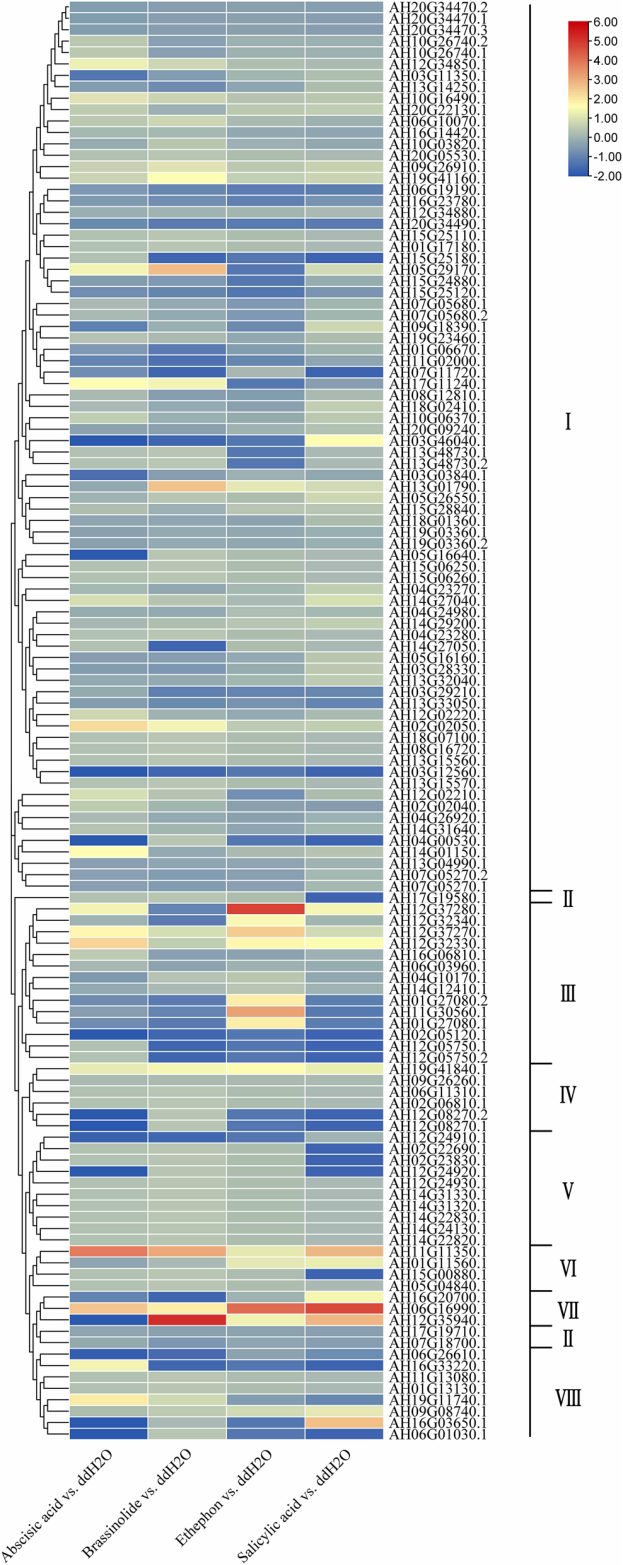
A heatmap showing the hierarchical clustering of the expression levels of the 126 *AhLEA*s under different hormone treatments in peanuts. I: *LEA2*; II: *Dehydrin*; III: *LEA3*; IV: *SMP*; V: *LEA5*; VI: *PvLEA18*; VII: *LEA4*; VIII: *LEA1*

To understand the expression changes of the *AhLEA*s under different hormones, the responses of 126 *AhLEA*s to four stress-related hormones (abscisic acid, brassinolide, ethylene, and salicylic acid) were investigated (Fig. 12). The expression profiles of these genes were examined by using transcriptomic data. After ABA treatment, 8 *LEA*s were induced more than twofold, while 19 *LEA*s were down-regulated more than twofold. After brassinolide treatment, 5 genes were up-regulated more than twofold, while and 31 genes were down-regulated more than twofold. The transcription of 13 *AhLEA*s was up-regulated more than twofold after ethylene treatment, while 28 genes were down-regulated more than twofold. The transcription of 10 *AhLEA*s was up-regulated more than twofold after salicylic acid treatment, while 16 genes were down-regulated more than twofold. Although the main *AhLEA*s were down-regulated by these four hormones, half of the *LEA3*s (7 out of 14) were up-regulated more than twofold after ethylene treatment, and all members of *LEA4*s were induced by salicylic acid. Moreover, the transcription of five *AhLEA*s was up-/down-regulated more than twofold by all four tested hormones. These genes included four *LEA2*s (AH06G19190.1, AH16G23780.1, AH20G34490.1, and AH16G06810.1) which were down-regulated after hormone treatment and a *PvLEA18* (AH11G11350.1) that was up-regulated (Additional file 1: Table S7).

### Expression pattern of *AhLEA*s under Al stress

**Fig. 13:**
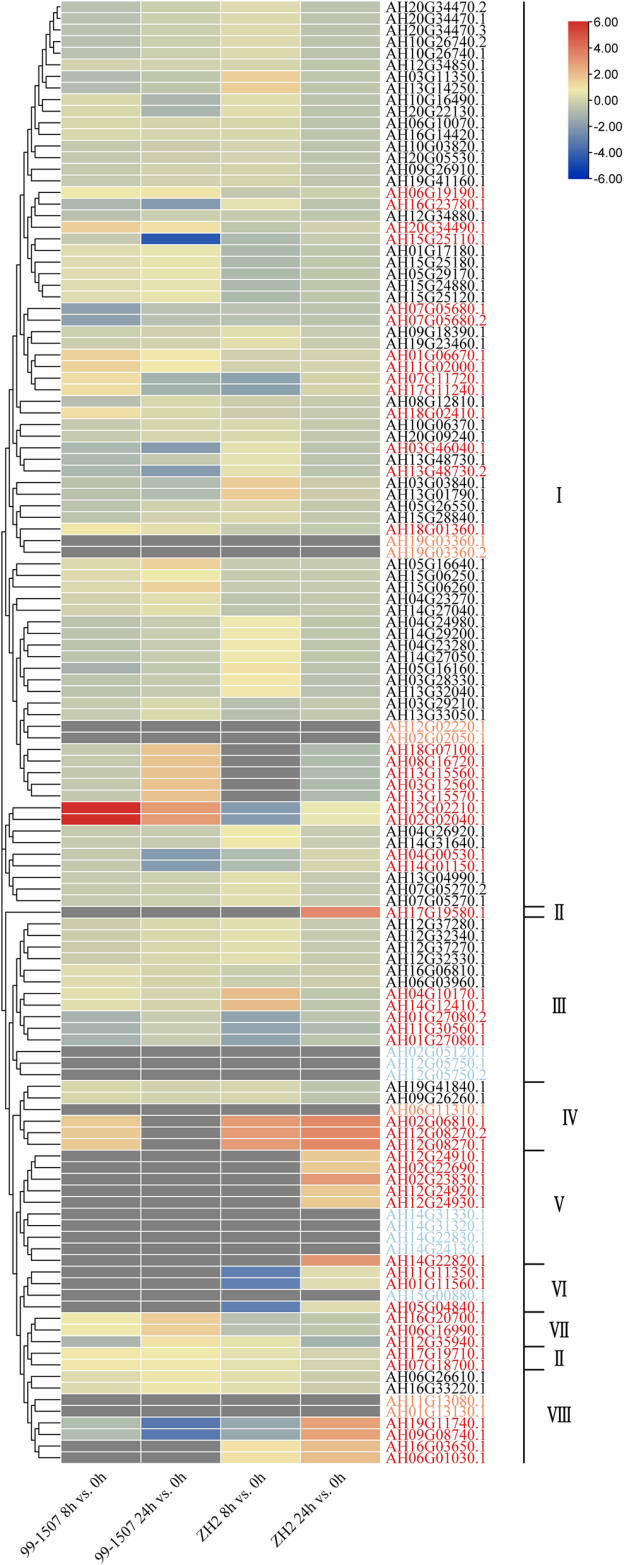
Expression profiles of Al-responsive *AhLEA*s in two varieties. The blue words represent not detected in the RNA-Seq dataset, and the orange words represent *LEA* genes were not expressed, the red words represent 50 differentially expressed genes. I: *LEA2*; II: *Dehydrin*; III: *LEA3*; IV: *SMP*; V: *LEA5*; VI: *PvLEA18*; VII:* LEA4*; VIII: *LEA1*

To gain a broader understanding of the putative functions of peanut *LEA*s in response to Al stress, the expression profiles of these genes were examined by using the RNA-Seq data which was generated from the root tips of two peanut cultivars that exhibited different Al sensitivity and had already been deposited in NCBI [24]. ZH2 is known as an Al sensitive peanut cultivar and 99–1507 is proved as an Al tolerant peanut cultivar [25]. Here, a total of 50 *AhLEA*s were found to be aluminum stress-responsive genes (Fig. 13, Additional file 1: Table S8). *LEA2*s which included twenty-three DEGs had the most aluminum stress-responsive genes. All of the members in *LEA4*s and *Dehydrin*s were aluminum stress-responsive genes, and both of these two subfamilies were composed of three genes. The aluminum stress-responsive genes accounted for 75% (3 out of 4), 60% (6 out of 10), 50% (3 out of 6, and 4 out of 8), and 35.7% (5 out of 14) of the members in *PvLEA18*s, *LEA5*s, *SMP*s (and *LEA1*s), and *LEA3*s, respectively.

Five *AhLEA*s were significantly up-regulated after 8 h of Al treatment in ZH2. Seventeen *AhLEA*s were significantly up-regulated after 8 h of Al treatment in 99–1507, including 66% of *LEA4*s and *Dehydrin*s (2 out of 3) and 50% of *SMP*s (3 out of 6). Nineteen *AhLEA*s were significantly up-regulated after 24 h of Al treatment in ZH2, including all members of *LEA5*s and *PvLEA18*s and half of *LEA1*s (4 out of 8) and *SMP*s (3 out of 6). Nine *AhLEA*s were significantly up-regulated after 24 h of Al treatment in 99–1507. Twelve *AhLEA*s were down-regulated after 8 h of Al treatment in ZH2, and all *PvLEA18*s were down-regulated. Two *AhLEA*s were down-regulated after 8 h of Al treatment in 99–1507. Seven *AhLEA*s were down-regulated after 24 h of Al treatment in ZH2. Eight *AhLEA*s were down-regulated after 24 h of Al treatment in 99–1507.

**Fig. 14:**
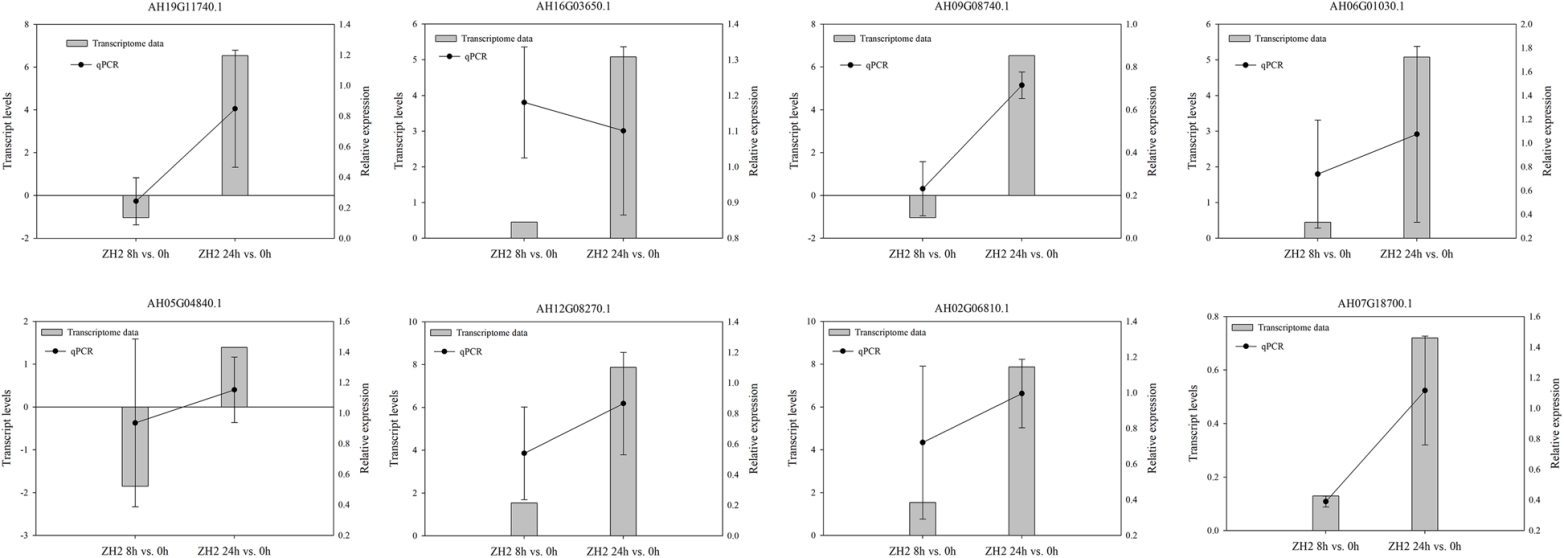
Relative expressions of *AhLEA*s under Al stress condition. The relative expression levels were calculated via the 2^–ΔΔCt^ method with the *AhACTIN* gene as an internal control. The error bars were created from three independent biological replicates

As ZH2 is a widely used commercial variety, eight differentially expressed *LEA* genes that were up-regulated greatly in ZH2 were selected for further qPCR analysis. As shown in Fig. 14, except for AH16G03650.1, the qPCR expression trends of the remaining seven genes were consistent with the transcriptomic data and were up-regulated after 24 h of aluminum treatment.

### *AhLEA*s overexpression enhanced *saccharomyces cerevisiae* BY4741 growth under drought and Al stress

Furthermore, we compared the DEGs in Al stress and the genes that were up-/down-regulated more than twofold under drought and low-temperature stresses. As shown in Fig S1, a total of 100 *AhLEA*s were regulated under drought, low temperature, and Al stresses. Among these genes, 35 common *AhLEA*s were involved in the responses to low-temperature and drought stresses, 29 common *AhLEA*s that were involved in the responses to drought and Al stresses, and 22 common *AhLEA*s that were involved in the responses to low-temperature and Al stresses. Sixteen *AhLEA*s were overlaps among the three abiotic stresses (Additional file 2: Fig S1).

To investigate the potential function of *AhLEA*s under stress conditions, the CDS sequence of three *LEA* genes, *AhLEA1*, *AhLEA3*-*1*, and *AhLEA3*-*3*, which were regulated under drought, low temperature, and Al stress were cloned. The function of three *AhLEA*s under these stresses was further investigated in eukaryotic cells as described by Gao [26].

**Fig. 15:**
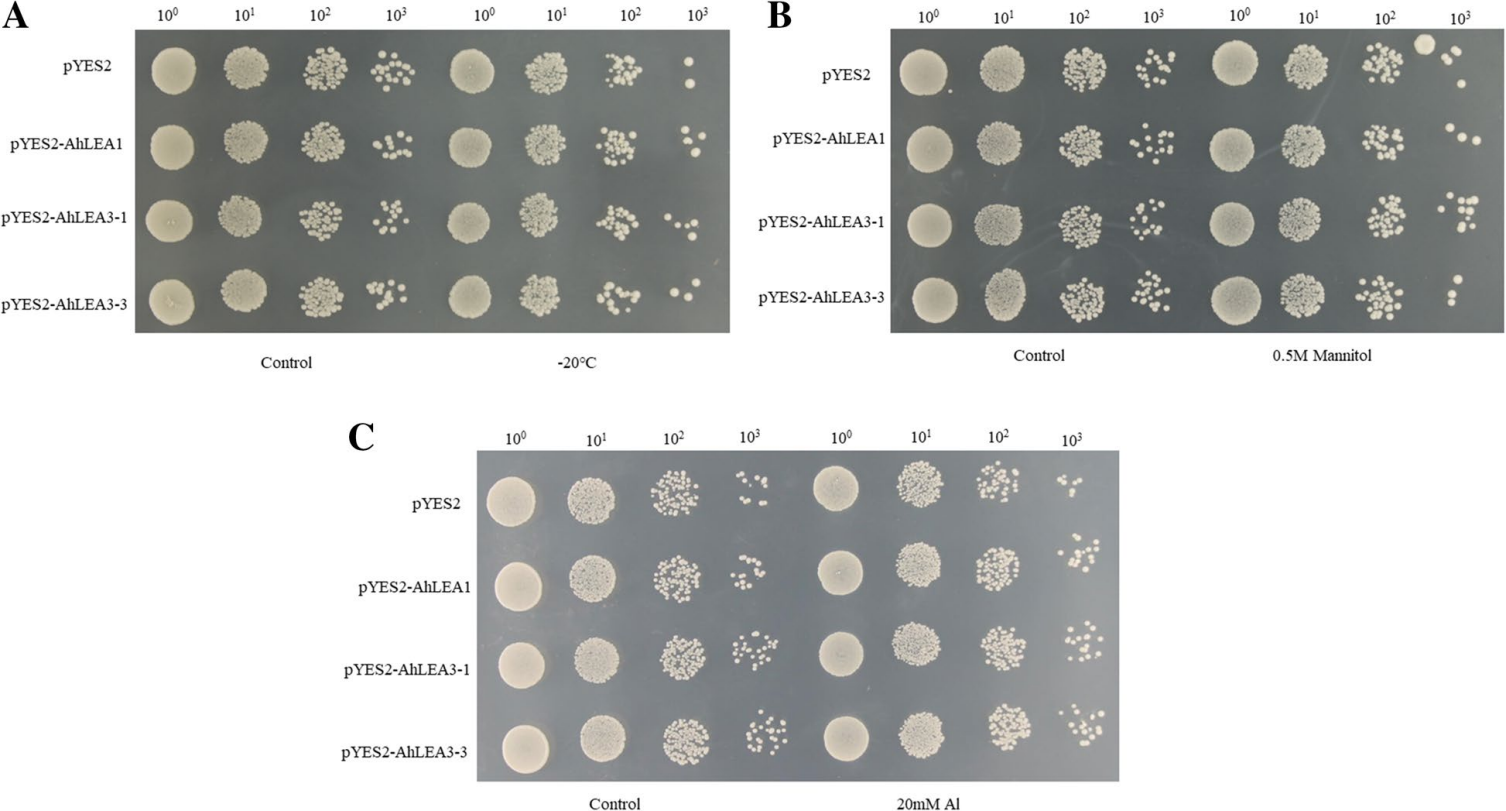
Growth of transformed yeast containing the pYES2-AhLEAs and pYES2 vectors under cold stress (A), drought stress (B), and Al stress (C). Note: Yeast cultures were grown in serial dilutions on SD-URA solid medium under control, -20 ℃ stress for 1 h, and 0.5 M Mannitol stress and 20 mM Al stress for 5 h

To assess the effect of *AhLEA1*, *AhLEA3*-*1*, and *AhLEA3*-*3* on the growth of recombinant yeast under freezing, drought, heat, NaCl, and Al stresses, BY4741 yeast containing pYES2- AhLEA1, pYES2- AhLEA3-1, pYES2- AhLEA3-3 and pYES2 vectors were subjected to -20 ℃, mannitol, 50 ℃, NaCl, and Al treatment, respectively. As shown in Fig. 15, under normal conditions, there was no significant difference between BY4741 that carrying recombinant plasmid pYES2- AhLEAs and empty vector pYES2. However, under Al and freezing (-20 ℃) stresses, BY4741 harboring pYES2- AhLEA1, pYES2- AhLEA3-1, or pYES2- AhLEA3-3 showed high viability than the yeast containing empty vector pYES2. In addition, BY4741 harboring pYES2- AhLEA3-1 had higher viability under drought stress compared to empty vector pYES2, while the yeasts containing pYES2- AhLEA1 or pYES2- AhLEA3-3 exhibited similar viability with the empty vector control (Fig. 15). This implies that heterologous expression of *AhLEA1*, *AhLEA3-1* and *AhLEA3-3* enhanced tolerance to cold and Al stresses in yeast, and *AhLEA3-1* could enhance the drought stress tolerance in yeast. Besides, under the high temperature condition of 50 ℃ and salt (NaCl) stresses, BY4741 harboring pYES2- AhLEA1, pYES2- AhLEA3-1, pYES2- AhLEA3-3 showed weaker viability with the yeast harboring empty vector pYES2 (Additional file 2: Fig. S2).

## Discussion

### Identification of the *LEA* gene family

In this study, 126 *LEA* genes were identified from whole peanut genome sequences. Based on the phylogenetic relationship with Arabidopsis, these 126 *AhLEA*s were distributed to eight groups. The number of peanut *LEA*s was twice that in Arabidopsis. According to the studies of the *LEA* family in other species, the number of *LEA*s may be related to the polyploidy of plants [27]. For example, many *LEA* genes were found in polyploids of *upland cotton* (Fang and Magwanga, 2018 [28]), *Triticum aestivum* [12]*,* and *Brassica napus* [11]. The *LEA2*s had more members than other subfamilies in the majority of species so far studied [27]. The *LEA2*s were the largest *LEA* subfamily in *Citrus sinensis, Oryza sativa, Populus trichocarpa* [29]*,* and *upland cotton* (Fang and Magwanga, 2018 [28]). Similarly, the *AhLEA*s mainly belong to the *LEA2*s, which accounted for 61.9% of the *LEA* genes. However, the *LEA2*s were not found as a large subfamily in the previous works in Arabidopsis [21]*, Oryza sativa* [30]*,* and *Populus trichocarpa* [5]. This result can be partly explained by the fact that improved annotation of higher plant genomes can be found on phytochrome (v10.2), and LEA2 is an unusual component of "a typical" LEA proteins because they are more hydrophobic. In addition, there were three pairs of tandem duplication in *AhLEA*s, which belong to the *LEA2*s. This result supported the view that tandem duplications have contributed significantly to the expansion and diversity of the *LEA2*s in most species [31].

### Analysis of conserved domains and introns revealed that *LEA*s might be stress-response genes

Motif analysis of the *AhLEA*s showed that members of each *LEA* group contained specific conserved motifs. Most members of the same subfamily have similar motifs, indicating an important role of these conserved motifs in the evolution of the *LEA* gene family. Big differences were found in the structure of different clades. For example, *LEA1*s contained motifs 13 and 17, whereas *LEA5*s contained motifs 2 and 9, which indicated the complexity and group-specific of LEA protein function. The conserved motifs observed in each *LEA* group suggested that genes with the same motif might be amplified from genes within the same evolutionary clade or group. It has been reported that stress-responding genes usually contain fewer introns (Fang and Magwanga, 2018 [28]; [32]. Introns have harmful effects on gene expression by delaying transcription products [33]. Moreover, introns can extend the length of new transcripts, resulting in additional energy consumption for transcription [34]. Up to 85.7% of the *AhLEA*s had zero or only one intron, which further suggested that *AhLEA*s were stress-related genes.

### Segmental duplication plays an important role in the evolution and amplification of *AhLEA*s

Gene duplication plays an important role in the evolution and amplification of gene families [35]. In this study, 93 pairs of segmental duplication and 5 pairs of tandem duplication were identified, it could be inferred that segmental duplication and tandem duplication contribute to the common expansion of the *AhLEA*s family, but the former played a predominant role. This finding was similar to previous studies on *Brassica napus* and upland cotton (*Gossypium hirsutum*) [11], Fang and Magwanga, 2018 [28]) and consistent with our previous study on receptor-like protein kinase (RLK) in peanut [36]. According to Ka/Ks estimation, 94.9% of the duplication gene pairs of *AhLEA*s were less than 1, indicating the results of the purification selection. The Ka/Ks ratios of two gene pairs (AH01G27080.1 and AH11G30560.1, AH05G16640.1 and AH15G06250.1) were more than 1, which indicated that these genes were in a state of positive selection in peanuts. We calculated the divergence time, and the results showed that many duplication events appeared to have occurred during relatively recent key periods. For example, all tandem duplication events occurred at 0–10 MYA, and 49.5% of segmental duplication occurred at 0–5 MYA. These results indicated that many *AhLEA*s were produced by the recent gene duplication events in peanuts. This may be related to the origin of cultivated peanut, through a single and recent polyploidization event, and then continuous selection in breeding work, forming a highly conserved genome [37].

The closer the species are, the greater the genome coverage of synteny fragments and the more genes they contain [38]. Syntenic analysis showed that more homologous gene pairs were found between peanut and soybean. There were 13 single peanut-to-Arabidopsis *LEA* gene correspondences and 5 single peanut-to-soybean *LEA* gene correspondences. These results indicated that these genes come from a common ancestor. Among these genes, two soybean genes (Glyma11g02290.1 and Glyma09g30400.1) did not belong to the *LEA* family. The rest genes showed one-to-more, more-to-one and more-to-more correspondence, and most of the genes included in these cases appeared more than once. However, 15 of the 23 orthologs of *AhLEA*s in soybean (Glyma09g38980.1, Glyma19g37350.1, Glyma08g22050.1, Glyma12g09590.1, Glyma12g32090.1, Glyma13g38380.1, Glyma03g34670.1, Glyma10g07360.1, Glyma13g21240.1, Glyma19g37340.1, Glyma06g01170.1, Glyma07g06960.1, Glyma13g43610.1, Glyma09g30400.1, and Glyma20g35880.1) were not *LEA* genes, which implied that their genomes underwent multiple rounds of chromosomal rearrangement and fusions. Allotetraploid‐cultivated peanut composed of A and B genomes and was generated from diploid *A. duranesis* (AA) and *A. ipaensis* (BB) [39]. Taking into account the divergence time of the duplications, we inferred that the divergence of many *AhLEA*s duplications occurred after the divergence of peanut and Arabidopsis/soybean from their last common ancestor. Combined with the results of phylogenetic tree analysis, there were nine orthologs including nineteen peanut *LEA* genes (AH12G35940.1-AT2G36640.1, AH17G19580.1-AT2G21490.1, AH12G35940.1-AT3G22500.1, AH02G22690.1/ AH12G24910.1-AT3G51810.1, AH05G04840.1/ AH15G00880.1-AT2G23110.1, AH04G10170.1/ AH14G12410.1-AT4G15910.1, AH06G03960.1/ AH12G32330.1/ AH12G37270.1/ AH16G06810.1-AT1G02820.1/ AT4G02380.3, AH02G06810.1/ AH12G08270.2-AT1G03120.1/ AT3G22490.1, AH02G02040.1/ AH04G26920.1/ AH12G02210.1/ AH14G31640.1-AT2G46140.1/ AT1G01470.1) that could be clustered together in the phylogenetic tree and were also contained in the syntenic map. We speculated that the functions of these *AhLEA*s were more similar to their Arabidopsis homologs than the other *AhLEA*s in the phylogenetic tree and syntenic map.

### MYB and MYC recognition sites may be involved in the response of *AhLEA*s to abiotic stress

Many studies have shown that *LEA*s play an important role in abiotic stress. In this study, many cis-acting elements related to abiotic stress and plant hormones were identified, such as ABRE, ERE, MYB recognition sites, MYC recognition sites, and STRE. We found that the MYB and MYC recognition sites were presented in the most promoters of the *AhLEA*s.

It is reported that MYBs and MYCs are transcription factors that participate in ABA-dependent signaling pathways to cope with abiotic stresses such as drought, salt, and low-temperature [40], Boter, 2014). Consistently, the *LEA*s that contain MYB recognition sites and MYC recognition sites, including AH16G06810.1, and AH06G03960.1, were induced under ABA, salicylic acid, drought, and low-temperature stresses. Besides, most of the *LEA1*s, *LEA5*s, *SMP*s, and *Dehydrin*s were highly expressed under aluminum stress, and these genes contained a large number of MYB and MYC recognition sites. Therefore, we speculated that the up-regulation of *LEA*s expression under aluminum stress might be regulated by MYB and MYC transcription factors. This provides a theoretical basis for further exploring the response regulation mechanism of *LEA*s containing cis-acting elements of MYB and MYC recognition sites under stress.

The plant hormone abscisic acid (ABA) accumulates mainly in leaves in response to drought stress, and ABA mediates various gene expression processes by stress responsive transcription factors (eg. ABREs, ABFs) [41]. Here, it was found that 28 *AhLEA*s contained ABRE cis-acting elements, of which 18 were in the *LEA2* family and 4 belonged to the *LEA3* family. Notably, seven genes had high transcription levels in response to drought stress. Previous studies found that the transcription levels of *LEA* genes were significantly up-regulated in root and shoot tissues after drought or ABA treatment [42]. These results suggested that *AhLEA*s responded to abiotic stresses such as drought, low-temperature, and Al stress might be activated directly or indirectly by ABA-dependent signaling pathways. Taken together, we proposed that most *LEA* genes have positive roles in coping with drought stress and that the seven genes containing ABRE cis-acting elements may be a direct target for ABA and have potential application value in improving drought tolerance in crops.

### Expression analysis revealed *AhLEA*s respond to different abiotic stresses

It can obtain clues from gene expression patterns to explore the function of genes [43]. We investigated the transcription level of *AhLEA*s in different tissues, at different embryo development stages, under different abiotic stresses (drought, low-temperature, and Al treatment), and after different hormone treatments. In four different embryo development stages, there were sixty-eight differentially expressed genes. Consistent with previous studies [10] that *LEA*s were up-regulated as the embryo developed, most of the *AhLEA*s were expressed at a high level at stages III and IV. However, the majority of *LEA3*s were highly expressed at an early stage, suggesting the potential roles of *LEA3*s in the early embryo development stage. As shown in Fig. 9, subfamily *LEA2* was the biggest subfamily, but the transcription levels of most *LEA2*s at four embryo development stages were stable, suggesting that *LEA2*s might not play important roles during embryo development.

The transcription level of most *AhLEA*s in the root, stem, leaf, and flower tissues was similar. The transcription level of many *AhLEA*s was low, while there were still several genes of subfamily *LEA2*, *LEA3*, and *Dehydrin* that exhibited a high transcription level in the four tissues. Two *LEA3*s (AH16G06810.1, AH06G03960.1) were very highly expressed in different peanut tissues (Fig. 10). It was reported that the *LEA3*s play an important role in plant growth, development, and response to abiotic stresses [44–46], and these two genes might be suitable candidates to understand the role of *LEA3*s in peanut.

Under drought stress, 50% of the *AhLEA*s were up-/down-regulated for more than twofold compared with control. Among them, *LEA2*s contributed most genes, containing 10 up-regulated genes and 24 down-regulated genes. This is consistent with the fact that *LEA2*s were the largest subfamily in peanuts. Among the genes that were down-regulated for more than twofold, most of them were *LEA2*s. Additionally, four *AhLEA1*s and three *AhLEA3*s were induced more than 60-fold by drought stress, implying their potential roles in enhancing drought stress tolerance in peanuts.

Under low-temperature stress, 36 *AhLEA*s were up-regulated more than twofold, while 18 genes were down-regulated more than twofold. *LEA2*s also contributed to most genes. Twenty-one *AhLEA2*s were up-regulated and eleven genes were down-regulated. Interestingly, the *LEA2*s that down-regulated under drought stress was also down-regulated under low-temperature stress, which suggested that there was a common mechanism to regulate *LEA2*s expression.

Many studies have been conducted to estimate the function of the *LEA* gene under abiotic stress in yeast [15, 26, 45–48]. For example, the overexpression of *TaHVA1*, tomato *le4*, *ZmLEA3* and *CpLEA5* improved the tolerance to low-temperature in yeast [26, 49]. Consistently, in our study, three *AhLEA*s (*AhLEA1*, *AhLEA3-1*, and *AhLEA3-3*) were found to enhance the cold stress tolerance in yeast. An important feature of LEA protein is its low molecular weight, which is a key factor in cell protection. Therefore, it is reasonable to speculate that the protective effect of *AhLEA1*, *AhLEA3-1*, and *AhLEA3-3* on cells under cold stress may be closely related to its low molecular weight and highly hydrophilic properties [50].

Seventeen genes were up-regulated after 8 h of Al treatment in 99–1507, and two of their (AH16G20700.1 and AH06G16990.1) were also up-regulated after 24 h of Al treatment. In ZH2, only five *AhLEA*s were up-regulated after 8 h of Al treatment, while sixteen *AhLEA*s were up-regulated after 24 h of Al treatment. Interestingly, three *SMP*s (AH12G08270.1, AH12G08270.2, and AH02G06810.1) were up-regulated after 8 h of Al treatment in both cultivars, suggesting that these genes might play important roles in Al tolerance in peanuts. Together, the Al-tolerant cultivar 99–1507 exhibited a rapid response to Al treatment, and the *LEA*s that induced rapidly should be studied in future work.

As shown in Fig. S1, the majority of the 126 *LEA*s were induced under at least one stress condition. Among these genes, sixteen were induced only under drought stress, fourteen were induced only under low-temperature, and sixteen were induced only under Al stress (Additional file 2: Fig S1). These results implied that these genes play distinct roles in response to different abiotic stresses in peanuts.

Some *AhLEA*s were regulated by different stress conditions. Three genes including two *LEA5*s (AH12G24910.1 and AH12G24920.1) and one *LEA1* (AH19G11740.1) were up-regulated greatly under both drought and Al stresses (Additional file 1: Table S6, Table S8). The expression of *LEA1* (AH19G11740.1) was induced more than twofold by ABA treatment. Two *LEA3*s (AH01G27080.1 and AH11G30560.1) and one *LEA4* (AH12G35940.1) were down-regulated under Al stress. The expression of that two *LEA3*s was significantly induced by ethylene, while *LEA4* (AH12G35940.1) was down-regulated by ABA treatment. Two *LEA2*s (AH02G02040.1 and AH12G02210.1) were up-regulated under drought, low-temperature, and Al stresses, and they were also up-regulated by ABA. The genes that respond to many stress conditions suggested a common regulation mechanism that plants adopted to cope with environmental challenges.

Many *AhLEA*s that were regulated more than twofold by hormones such as abscisic acid, brassinolide, ethylene, and salicylic acid were found to be down-regulated. As revealed by table S7, these down-regulated genes showed no obvious subfamily preference. However, *AhLEA*s that were up-regulated more than twofold by ethylene and salicylic acid showed obvious subfamily preference. Seven *AhLEA3*s were induced by ethylene. Five *AhLEA3*s induced by ethylene were also involved in response to drought and low-temperature stresses. The transcription level of AH12G37280.1 was increased up to 8.45-fold under low-temperature stress. AH12G32330.1 was up-regulated 3.5-fold under drought stress. Moreover, three *AhLEA3*s (AH01G27080.1, AH01G27080.2, AH11G30560.1) were up-regulated greatly under both drought and low-temperature stresses. These results revealed the important roles of the *AhLEA3* subfamily in the ethylene-mediated response under drought and low-temperature stresses. Additionally, all *AhLEA4*s were induced by salicylic acid, and all *AhLEA4*s were also regulated greatly under drought and low-temperature stresses. Among them, two genes (AH06G16990.1 and AH12G35940.1) were induced more than sixfold under drought and low-temperature stresses, and one gene (AH16G20700.1) was down-regulated 3.5-fold under low-temperature stress, which implied that subfamily *AhLEA4* played important roles in SA-mediated response under drought and low-temperature stresses in peanut.

Taken together, these results suggested that common mechanisms might be initiated in peanuts to cope with different abiotic stresses. Hormones were involved in regulating *LEA*’s expression under abiotic stresses. The role of hormones in regulating gene expression had a preference among *AhLEA* gene families.

## Conclusions

In this study, 126 *LEA* genes in *Arachis hypogaea* were identified. They were divided into eight groups according to homologous in *Arabidopsis thaliana*. *AhLEA*s are randomly distributed on the chromosome, and most of them may be segmental duplication. The exon–intron and motif structures indicated that the *LEA*s’ family functions were highly conserved. Some cis-elements of abiotic stress response were also found in the upstream sequences of most *AhLEA*s. The comprehensive analysis of *AhLEA*s gene expression profiles showed that the *LEA3*s, *LEA4*s, and *SMP*s played an important role in abiotic stress response, and also showed the functional differences among other subfamilies. Moreover, the functions of AhLEA1, AhLEA3-1 and AhLEA3-3 proteins were verified and found to enhance cold and aluminum tolerance in yeast, and AhLEA3-1 enhanced the drought tolerance in yeast. This study provided a reference for further exploring the mechanism of *LEA*s in response to abiotic stress in peanuts.

## Materials and methods

### Identification of *LEA*s in peanut

To identify the *AhLEA*s, we used 51 *LEA* genes [21] in *Arabidopsis thaliana* acquire Pfam ID (PF03760, PF03168, PF03242, PF02987, PF00477, PF10714, PF04927, PF00257) and InterPro ID (IPR005513, IPR004864, /IPR013990, IPR004926, IPR004238, IPR000389, IPR018930, IPR007011, IPR000167) from Peanut Base (https://www.peanutbase.org/). By acquiring LEA peanut protein sequences based on InterPro ID search of Peanut Genome Resource (PGR) (http://peanutgr.fafu.edu.cn/). NCBI’s Conserved Domains Database (https://www.ncbi.nlm.nih.gov/cdd) and PFAM (http://pfam.xfam.org/) database were used to verify the presence of the *LEA* domains and finally obtained 126 *AhLEA*s.

### Phylogenetic relationships, gene structures, conserved motifs, and chromosomal locations of the *AhLEA*s

The phylogenetic tree was constructed by the maximum-likelihood method with 1000 bootstrap replicates in MEGA 7.0 software [51]. Multiple Expectation Maximization for Motif Elicitation (MEME) (http://meme-suite.org/tools/meme) [52] was used to identify the conserved protein motifs, with a maximum number of the different motif at 20. The exon–intron structures were identified using the TBtools software [53]. The physical location of each *AhLEA* is determined by identifying the starting position of all genes on each chromosome, searching the local database of Peanut Genome Resources by BLAST. Using TBtools of Gene location visualize from GFF/GFF3 to draw chromosome mapping and tandem duplication pairs.

### Promoter cis-element analysis

Genomic data were obtained from Peanut Genome Resource (PGR) (http://peanutgr.fafu.edu.cn/), and TBtools software was used to extract all *LEA* upstream 2kd promoter sequences. Transcriptional response elements of *LEA* gene promoters were predicted using the PlantCARE database (http://bioinformatics.psb.ugent.be/webtools/plantcare/html/) [54].

### Gene duplication and evolutionary analysis

We used Virtual Machine to construct the tandem and segmental of the putative duplication of the *AhLEA*s and calculate the ratio of the nonsynonymous substitution rate (Ka) to the synonymous substitution rate (Ks) by the Simple Ka/Ks calculator (NG) of TBtools [53]. *LEA*s clustered together within 100 kb, length of the alignable sequence covers > 75% of longer gene and similarity of aligned regions > 75% were regarded as tandem duplicated genes. The relationship between Ka/Ks ratio and value 1, Ka larger than Ks (or Ka/Ks >  > 1), Ka equals Ks (Ka/Ks = 1), and Ka less than Ks (or Ka/Ks <  < 1), which represent positive (or diversifying) selection, neutral evolution and negative (or purifying) selection, respectively. Divergence time was calculated with the formula T = Ks/2r, where r is 1.5 × 10^−8^ synonymous substitutions per site per year and it is the rate of divergence for nuclear genes from plants [55]. We used Multiple Synteny Plot software [53] to explore the collinear relationship between the *AhLEA* and *LEA* genes from *Arabidopsis thaliana* and *Glycine max*. All the soybean LEA domain-containing protein sequences were downloaded from the Soybase Glyma.Wm82.a2.v1 (http://www.soybase.org/). The NCBI’s Conserved Domains Database (https://www.ncbi.nlm.nih.gov/cdd) and PFAM (http://pfam.xfam.org/) database were used to verify the presence of the LEA domains. The *GmLEA*s that were identified in the previous study were also screened [56]. After eliminating the invalid sequence, a total of 132 *GmLEA*s were identified.

### Expression analysis of *AhLEA*s

The blast was performed in the transcriptome of the PGR database using the protein sequences of 126 *AhLEA*s. RNA-Seq data were downloaded from PGR (http://peanutgr.fafu.edu.cn/Download.php) and used to generate the expression patterns of *AhLEA*s in different tissues (root, stem, leaf, and flower), different embryo development stages, and various abiotic stresses (cold, and drought), and different hormones treatment on leaves. Transcriptome data that were generated from peanut root tips under Al stress were used to generate the expression patterns of *AhLEA*s under Al stress. The data had been deposited in the database of the National Center for Biotechnology Information (NCBI) under accession number PRJNA525247 (https://www.ncbi.nlm.nih.gov/sra/PRJNA525247). TBtools were used to generate heat maps and combine phylogenetic tree, gene, and protein structure [53].

### The expression of *AhLEA*s in *Saccharomyces cerevisiae* BY4741

According to the full-length coding sequence of the *AhLEA1*, *AhLEA3-1*, and *AhLEA3-3* in the peanut genome resource, specific primers (Additional file 1: Table S9) were designed using CE Design software. Using this primer to amplify *AhLEA*s from cDNA, and the purified PCR products were cloned into the *pMD19*-*T* vector (TaKaRa, Dalian, China) for sequencing (Aoke, China). The correct sequence was inserted into the intracellular expression vector pYES2/CT for *Saccharomyces cerevisiae*.

The pYES2-AhLEAs fusion protein was expressed in *Saccharomyces cerevisiae* BY4741. Yeast harboring pYES2-AhLEAs and pYES2 were incubated in SD-URA (2% Glucose) medium to OD_600_ = 0.6, and the yeast solution was added to SG-URA (2% Galactose) medium at a ratio of 20: 1 induce protein at 30 °C for 48 h. And then, 1 mL yeast culture was treated at 0.5 M mannitol, 0.5 M NaCl, 20 mM AlCl_3_ for 5 h, respectively. Similarly, 1 mL yeast culture was treated at –20 °C and 50 °C for 1 h, respectively. In addition, 1 mL yeast culture was taken out as normal condition control. Then, 10 µL yeast culture from different treatments at different dilution ratios (10°, 10^1^, 10^2^, and 10^3^) were dropped on SD-URA solid medium. After 48 h of culture at 30 °C, the growth situation of the yeast cells was observed and recorded as described in Gao’s (2020) report.

### QRT-PCR analysis of the *AhLEA*s

The experiment was carried out with peanut root tips, and the treatment method was referred to as our previous report [57]. The gene specific primers of *AhLEA*s were designed, and the *AhACTIN* was used as the reference gene (Additional file 1: Table S9). The qRT-PCR was performed using SYBR^Ⓡ^ Premix Ex Taq™ II (TaKaRa, Dalian, China). Three independent biological replicates were performed and the relative expression levels of *AhLEA* were calculated using the 2^−ΔΔCT^ method.

## Supplementary Information

**Additional file 1.**

**Additional file 2: Fig S1. **Venn diagram showing the number of AhLEAs that responded to drought, and lowtemperature, Al stresses. **Fig S2.** Growth of transformed yeast containing the pYES2-AhLEAs and pYES2 vectors under heat stress (A) and salt stress (B).

## Data Availability

All raw data is downloaded from the public databases. The RNA-seq data of ZH2 and 99–1507 under Al treatment had been deposited in the database of the National Center for Biotechnology Information (NCBI) under accession number PRJNA525247 (https://www.ncbi.nlm.nih.gov/sra/PRJNA525247). The raw RNA-seq reads in different peanut tissues and after different treatments (including different hormones, drought and low temperature stresses) are available at Peanut Genome Resource (http://peanutgr.fafu.edu.cn/Download.php), and *AhLEA*s sequences are available at Peanut Genome Resource (http://peanutgr.fafu.edu.cn/Transcriptome.php). All data generated or analyzed in this study are included in this published article [Additional file 1. xlsx]. The plant and yeast materials used in the current study are available from the corresponding author on reasonable request.

## Abbreviations

Al: Aluminum
Al stress: Aluminum stress
*At*: *Arabidopsis thaliana*
*Ah*: *Arachis* *hypogaea*. L
ABRE: ABA-responsive element
ERE: Ethylene response element
WRE3: Water response element
MYB: Transcription factor
MYC: Transcription factor
TC-rich repeats: Cis-acting element involved in defense and stress responsiveness
MRE: Metal responsive element
STRE: Stress response element
DEGs: Differentially expressed genes
