# Supplementary material for: Genome-wide identification, evolutionary and expression analyses of LEA gene family in peanut (Arachis hypogaea L.)
Source: BMC Plant Biol. 2022 Mar 30;22:155. doi: 10.1186/s12870-022-03462-7 (PMC8966313; doi:10.1186/s12870-022-03462-7)
Supplement: Supplementary file 2 — Additional file 2: Fig S1. Venn diagram showing the number of AhLEAs that responded to drought, and lowtemperature, Al stresses. Fig S2. Growth of transformed yeast containing the pYES2-AhLEAs and pYES2 vectors under heat stress (A) and salt stress (B). [file 12870_2022_3462_MOESM2_ESM.pdf]

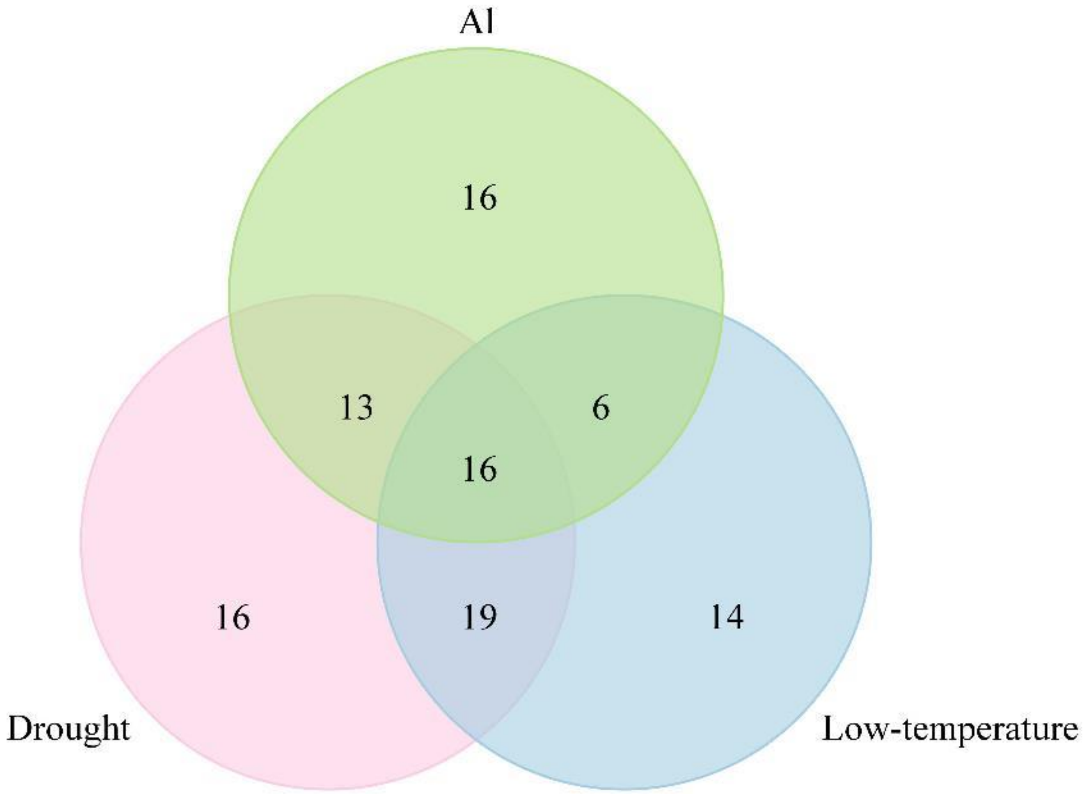

Fig S1: Venn diagram showing the number of *AhLEA*s that responded to drought, and low-temperature, Al stresses.

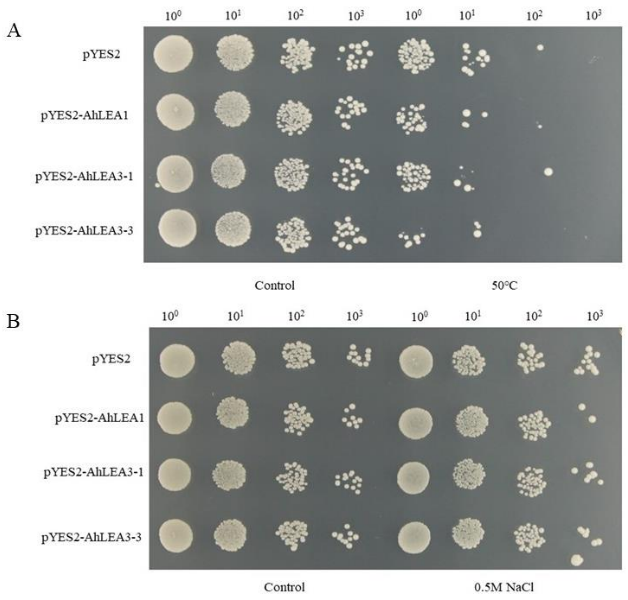

Fig S2: Growth of transformed yeast containing the pYES2-AhLEAs and pYES2 vectors under heat stress (A) and salt stress (B).

Note: Yeast cultures were grown in serial dilutions on SD-URA solid medium under control, 50 °C stress for 1h, and 0.5M NaCl stress for 5h.
